# Electron Beam Melting and Refining of Metals: Computational Modeling and Optimization

**DOI:** 10.3390/ma6104626

**Published:** 2013-10-18

**Authors:** Katia Vutova, Veliko Donchev

**Affiliations:** Institute of Electronics, Bulgarian Academy of Sciences, 72 Tzarigradsko shosse, Sofia 1784, Bulgaria; E-Mail: velikod@gmail.com

**Keywords:** computational modeling, heat model, computer simulation, thermal transfer, optimization, electron beam melting and refining, metals

## Abstract

Computational modeling offers an opportunity for a better understanding and investigation of thermal transfer mechanisms. It can be used for the optimization of the electron beam melting process and for obtaining new materials with improved characteristics that have many applications in the power industry, medicine, instrument engineering, electronics, *etc*. A time-dependent 3D axis-symmetrical heat model for simulation of thermal transfer in metal ingots solidified in a water-cooled crucible at electron beam melting and refining (EBMR) is developed. The model predicts the change in the temperature field in the casting ingot during the interaction of the beam with the material. A modified Pismen-Rekford numerical scheme to discretize the analytical model is developed. These equation systems, describing the thermal processes and main characteristics of the developed numerical method, are presented. In order to optimize the technological regimes, different criteria for better refinement and obtaining dendrite crystal structures are proposed. Analytical problems of mathematical optimization are formulated, discretized and heuristically solved by cluster methods. Using important for the practice simulation results, suggestions can be made for EBMR technology optimization. The proposed tool is important and useful for studying, control, optimization of EBMR process parameters and improving of the quality of the newly produced materials.

## 1. Introduction

**Figure 1:**
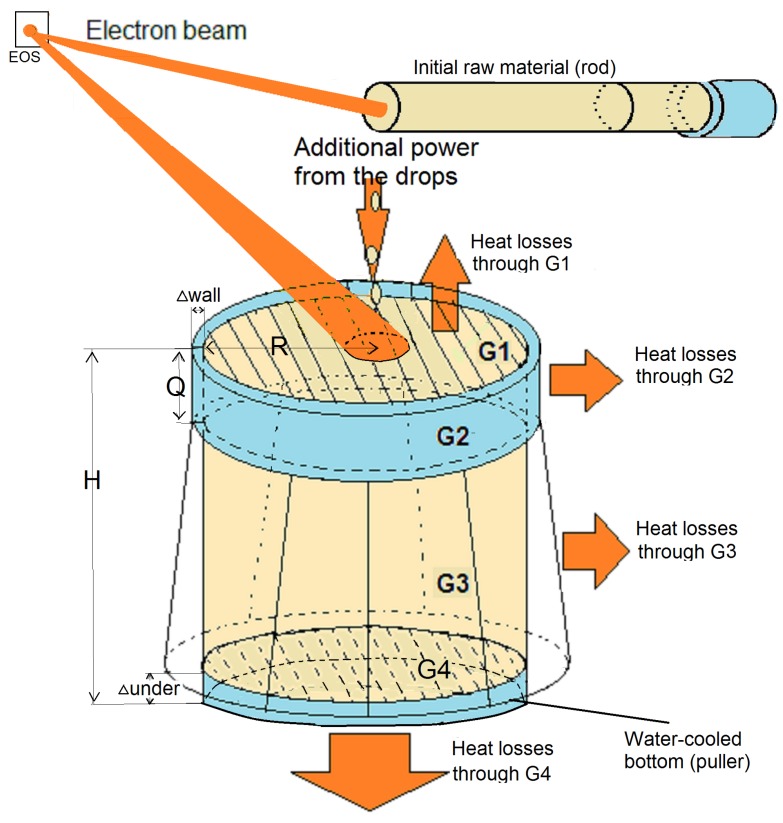
Scheme of Electron beam melting and refining (EBMR) process. *G1*—the top surface of the formed pure ingot; *G2*—the interface molten ingot/ water-cooled crucible side wall; *G3*—the interface ingot/ vacuum; *G4*—the interface ingot/water-cooled puller.

Electron beam melting and refining (EBMR) in a vacuum using an intense electron beam is a widely used, ecologically-friendly method in special electro metallurgy for new materials fabrication: the production of pure metals and special alloys [1,2,3,4,5,6]. The obtained materials have improved chemical compositions, structures, properties, and have many applications in medicine, aerospace engineering, nuclear industry, instrument engineering, *etc.* The electron beam melting and refining of metals is accomplished in a vacuum chamber [the working vacuum pressure is about (5–8) × $10^{-3}$ Pa] using electron beams as a heating source. The raw material is melted, refined and re-solidified in a water-cooled copper crucible, which is used due to the increasing demand for purity of the transformed materials (Figure 1). The electrons fall on the front side of the feeding rod (raw material) and heat it. Then drops of molten metal fall in the crucible. The top surface of the molten metal in the water-cooled crucible *(G1)* is also heated by the electrons (Figure 1). By using a water-cooled pulling mechanism (Figure 1), the operator can withdraw the bottom of the growing formed pure metal ingot. This allows the liquid pool surface to be maintained at a constant level suitable to be viewed by the operator. This method provides a high refining level (high level of impurities removal—gases, metals and non-metals), chemical composition homogeneity and optimal structure of the cast pure ingots [1,2,3,4,5,6].

Despite development of the technology, there are many unsolved problems concerning mechanisms and relationships of macroscopic heat and mass transfer during heating free liquid surface with intensive energy flow, that still exist [3,4,5,6,7]. For the EBMR process and equipment optimization and the price of the produced pure materials, the questions concerning the improved energy efficiency are crucial. The answer of these questions depends on the detailed study of the heat transfer processes taking place in the zone of interaction beam-material, on the processes and factors limiting the geometry of the molten pool and the precise evaluation of the temperature field dynamics and behavior of the metals and their compounds during the EBMR. Due to difficulties in acquiring real time information about the processes in the molten pool [1,2], the successful application and optimization of EBMR depends also on the adequate mathematical modeling of the processes that allow for studying the influence of many technological parameters and the various limiting factors. Stationary and quasi-stationary heat models in this field are already published [8,9,10,11,12,13,14,15] and reveal the importance of some process characteristics. In [16] a finite element based mathematical model to predict the evolution of the temperature and stress during EBMR of solar-grade silicon ingots is also presented. Applying a heuristic approach the flatness of the liquid/solid boundary is examined and studied [17,18].

A time-dependent thermal mathematical model of the electron beam drip melting and refining process is developed and described in this paper. The model is a continuation and an extension of our quasi-steady-state heat model [10,12,15]. The boundary conditions on the ingot interfaces are formulated assuming different mechanisms of the heat transfer. The temperature variations of the thermo-physical parameters (heat capacity, thermal conductivity, *etc*.) are taken into account in the heat model. An optimization scheme, based on the heat model, is proposed and applied for quality improvement of new materials obtained by EBMR.

## 2. Modeling and Mathematical Setting of the Problem

### 2.1. Main Equation

Let *H* be the ingot’s height, *R*—the ingot’s radius (Figure 1), and *F*—the maximal heating time that is chosen. The investigated temperature distributions are along cylindrical ingots (samples) and an assumption for angle symmetry is applied. Then in:

(1) $$\begin{array}{c} \Omega =\{x=(r,z,t)|0\leq r\leq R,0\leq z\leq H,0<t\leq F\} \end{array}$$

**Figure 2:**
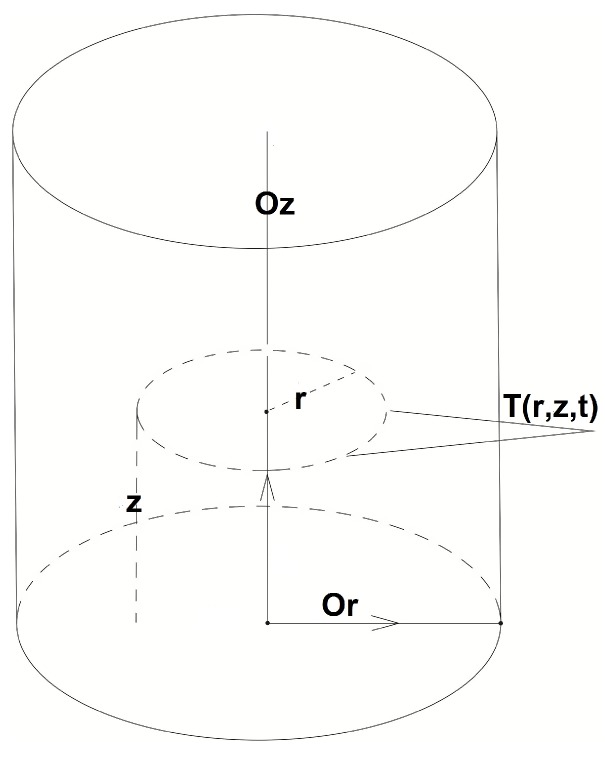
Cylindrical metal ingot, T(r,z,t)—the temperature at the moment *t* in the ingot’s point with coordinates (r,z).

T(r,z,t) denotes the temperature at time *t* at the points with height *z* and polar distance *r* (Figure 2). In the inner points of Ω, the temperature distribution T(x) is described by the heat Equation (2) in cylindrical coordinates:

(2) $$\begin{array}{c} \frac{1}{r}\frac{\partial }{\partial r}\left(r\frac{\partial T}{\partial r}\right)+\frac{\partial^{2}T}{\partial z^{2}}+\frac{V}{a}\frac{\partial T}{\partial z}=\frac{\rho C_{p}}{\lambda }\frac{\partial T}{\partial t} \end{array}$$

where *ρ* [kg/m${}^{3}$ ] is the density of the metal; $C_{p}$ [W · s/kg · K] is the heat capacity; *λ* [W/m · K] is the thermal conductivity; *a* [m${}^{2}$/s] is the thermal diffusivity and $a=\lambda /(\rho C_{p})=1/k$.

The last term in Equation (2) $\frac{V}{a}\frac{\partial T}{\partial z}$ shows the casting, *i.e.*, the heat added by the poured molten metal (from the melting raw material—Figure 1) into the crucible. It is given by the thermal energy transfer from the material moving with velocity *V*, coincident to the *z*-axis (Figure 1 and Figure 2). When V≠0, the model describes the solidification of the cast ingot in the water-cooled crucible—“drip melting process" (Figure 1), while the case V=0 describes the “disks melting method" (no material is added to the ingot by pouring) [2,10]. The thermo-physical parameters *λ* and $C_{p}$ of the investigated metals are modeled as functions of the temperature (by linear regression method) using experimental data [19,20,21,22].

### 2.2. Equations for the Boundary Conditions

The boundaries *G1, G2, G3, G4* (Figure 1) are described as follows:

(3) $$\begin{array}{cc} \Omega_{1} & =\{(r,H,t)|0\leq r\leq R,t\geq 0\} \end{array}$$

(4) $$\begin{array}{cc} \Omega_{2} & =\{(R,z,t)|H-Q\leq z\leq H,t\geq 0\} \end{array}$$

(5) $$\begin{array}{cc} \Omega_{3} & =\{(R,z,t)|0\leq z\leq H-Q,t\geq 0\} \end{array}$$

(6) $$\begin{array}{cc} \Omega_{4} & =\{(r,0,t)|0\leq r\leq R,t\geq 0\} \end{array}$$

The boundary Equations that correspond to these interfaces are:

(7) $$\begin{array}{c} \lambda \left(T\left(x\right)\right)\frac{\partial T}{\partial z}\left(x\right)=\left|+P_{surf}\left(r,t\right)-\alpha \sigma \left(T^{4}\left(x\right)-T_{room}^{4}\right)-C_{p}\left(T\left(x\right)\right)\cdot W_{v}\cdot T\left(x\right)\right|,x\in \Omega_{1} \end{array}$$

(8) $$\begin{array}{cc} \lambda \left(T\left(x\right)\right)\frac{\partial T}{\partial r}\left(x\right) & =\frac{\partial T^{1}}{\partial r}\left(x\right),x\in \Omega_{2} \end{array}$$

(9) $$\begin{array}{cc} \lambda \left(T\left(x\right)\right)\frac{\partial T}{\partial r}\left(x\right) & =-\alpha \sigma \left(T^{4}\left(x\right)-T_{room}^{4}\right),x\in \Omega_{3} \end{array}$$

(10) $$\begin{array}{cc} \lambda \left(T\left(x\right)\right)\frac{\partial T}{\partial z}\left(x\right) & =\frac{\partial T^{2}}{\partial z}\left(x\right),x\in \Omega_{4} \end{array}$$

The heat transfer through *G1* boundary (Equation (7)), where the radiation losses predominate, is described by the Stefan-Boltzmann law [3,12]. In Equation (7) *α* is the metal’s emissivity, $\sigma =5.6704\times 10^{-8}$ [J/s · m${}^{2}\cdot$ K${}^{4}$] is the Stefan-Boltzmann constant. $P_{surf}\left(r,t\right)$ is the e-beam power density function and usually Gaussian-like distribution is used [2], defined by the beam diameter’s value. The vapor losses are calculated by $C_{p}\cdot W_{v}\cdot T$ (Equation (7)). The weight loss velocity $W_{v}$ [kg/s · m${}^{2}$] is evaluated using experimental data for the material losses obtained during EBMR process; $T_{room}$ is the room temperature (300 K).

Equation (8) describes Newton’s type of heat transfer through *G2*—the interface molten metal/water-cooled crucible side wall. The gradient $\partial T^{1}/\partial r$ is equal to $\lambda_{2}\left(T_{water}-T\left(x\right)\right)/\Delta wall$; $\lambda_{2}$ is the conductivity of the crucible’s material (copper). When the liquid metal pool does not reach the interface *G2* and/or its volume is small this boundary condition can be neglected (*i.e*., Q=0, Figure 1). Δ wall is the width of the crucible side wall and $T_{water}$ is the mean water temperature in the water-cooling system, $(T_{water}=300$ K).

The thermal transfer through the interface ingot/ vacuum *(G3)* is described by the Stefan-Boltzmann law and is represented by Equations (9) and (10) describes the heat transfer through the ingot bottom *(G4)*—area with an ideal heat contact. The gradient $\partial T^{2}/\partial z$ is equal to $\lambda_{2}\left(T_{water}-T\left(x\right)\right)/\Delta under$, where Δunder is the width of the water-cooled puller (Figure 1).

The initial condition gives information about the temperature distribution at the moment t=0 s. Usually, the temperature along the metal ingot at the first moment is a constant and is equal to the mean room temperature $T_{room}$. This condition is described by Equation (11):

(11) $$\begin{array}{cc} T(x) & =T_{0}\left(r,z\right),x\in \Omega_{0} \end{array}$$

(12) $$\begin{array}{cc} \Omega_{0} & =\{(r,z,0)|0\leq r\leq R,0\leq z\leq H\} \end{array}$$

### 2.3. Heat Streams

The heat streams through the boundaries at the moment *t* are calculated as follows:

#### 2.3.1. Energy Losses (Radiation and Vapor Losses) through *G1*:

(13) $$\begin{array}{cc} S_{G1,r}\left(t\right) & =\int \;\;\;\int_{\left[0,2\pi ]\times [0,R\right]}\alpha \sigma \left(T^{4}\left(r,H,t\right)-T_{room}^{4}\right)rdrd\phi \end{array}$$

(14) $$\begin{array}{cc} S_{G1,v}\left(t\right) & =\int \;\;\;\int_{\left[0,2\pi ]\times [0,R\right]}C_{p}\left(T\left(r,H,t\right)\right)\cdot W_{v}\cdot T\left(r,H,t\right)rdrd\phi \end{array}$$

#### 2.3.2. Streams through the Boundaries *G2*, *G3* and *G4*:

(15) $$\begin{array}{cc} S_{G2}\left(t\right) & =\int \;\;\;\int_{\left[0,2\pi ]\times [H-Q,H\right]}\lambda \left(T\left(R,z,t\right)\right)\frac{\partial T}{\partial r}\left(R,z,t\right)Rdzd\phi \end{array}$$

(16) $$\begin{array}{cc} S_{G3}\left(t\right) & =\int \;\;\;\int_{\left[0,2\pi ]\times [O,H-Q\right]}\lambda \left(T\left(R,z,t\right)\right)\frac{\partial T}{\partial r}\left(R,z,t\right)Rdzd\phi \end{array}$$

(17) $$\begin{array}{cc} S_{G4}\left(t\right) & =\int \;\;\;\int_{\left[0,2\pi ]\times [0,R\right]}\lambda \left(T\left(r,0,t\right)\right)\frac{\partial T}{\partial z}\left(r,0,t\right)rdrd\phi \end{array}$$

#### 2.3.3. The Incoming Heat is Determined by the Heating Beam Energy Distribution $P_{surf}$ and by the Heat Added by the Poured Molten Drops $P_{add}$:

(18) $$\begin{array}{c} S_{p}\left(t\right)=\int \;\;\;\int_{\left[0,2\pi ]\times [0,R\right]}P_{surf}\left(r,t\right)rdrd\phi \end{array}$$

The additional heat due to the added liquid drops is calculated by:

(19) $$\begin{array}{c} P_{add}=\pi R_{rod}^{2}V\rho \left(C_{p}^{*}T_{melt}+q\right) \end{array}$$

where $R_{rod}$ is the radius of the feeding rod (Figure 1); *q* [W · s/kg] is the specific melting heat of the metal; $T_{melt}$ is the melting temperature of the investigated metal; $C_{p}^{*}$ is an average of the heat capacity. The heat transfer processes during EBMR become stationary when the difference between the input heat and total energy losses through the boundaries becomes close to 0.

## 3. Numerical Method

A modified Pismen-Rekford method [23] is developed and applied for solving the problem Equations (2) and (7–11) and for calculating the temperature fields in the cast ingot. A regular net of points $W_{h_{1},h_{2},\tau }$ in the domain Ω concerning Equations (2) and (7–11) is made:

(20) $$\begin{array}{c} W_{h_{1},h_{2},\tau }=\left\{\left(r_{i},z_{j},t_{n}\right)|r_{i}=ih_{1},z_{j}=jh_{2},t_{n}=n\tau ;i=\bar{0,N},j=\bar{0,M},n=\bar{0,P}\right\} \end{array}$$

$T_{i,j}^{n}$ denotes the approximated temperature in the point $\left(r_{i},z_{j},t_{n}\right)$ of the grid. Additionally, $T^{n}$ is the matrix of M×N points, corresponding to the temperature distribution at the moment $t_{n}=n\tau$. For the purposes of the algorithm intermediate planes $T^{n+1/2}$ (which are not included in the net $W_{h_{1},h_{2},\tau }$) are built. Approximations $T^{n}$ of T(r,z,τn), continuously starting from n=0 to n=P, are made. Geometrically, “jumps" are continuously made on rectangles $\Omega^{n}=\left\{\left(r,z,\tau n\right)|0\leq r\leq R,0\leq z\leq H\right\}$, parallel to $\Omega_{0}$. For each *n*, two jumps are associated and also two mathematical problems (A) and (B) have to be solved:

(21) $$\begin{array}{cc} \left(A\right)\;k\frac{T^{n+\frac{1}{2}}-T^{n}}{0.5\tau } & =\Lambda_{1}T^{n+\frac{1}{2}}+\Lambda_{2}T^{n} \end{array}$$

(22) $$\begin{array}{cc} \left(B\right)\;k\frac{T^{n+1}-T^{n+\frac{1}{2}}}{0.5\tau } & =\Lambda_{1}T^{n+1}+\Lambda_{2}T^{n+\frac{1}{2}} \end{array}$$

Here $\Lambda_{1}$ and $\Lambda_{2}$ are difference operators, corresponding to $\frac{1}{r}\frac{\partial }{\partial r}\left(r\frac{\partial T}{\partial r}\right)$ and $\frac{V}{a}\frac{\partial T}{\partial z}$, respectively:

(23) $$\begin{array}{cc} \Lambda_{2}T_{i,j}^{n} & =\frac{T_{i,j-1}^{n}-2T_{i,j}^{n}+T_{i,j+1}^{n}}{h_{2}^{2}} \end{array}$$

(24) $$\begin{array}{cc} \Lambda_{1}T_{i,j}^{n} & =\frac{T_{i-1,j}^{n}-2T_{i,j}^{n}+T_{i+1,j}^{n}}{2h_{1}^{2}}+\frac{1}{ih_{1}}\frac{T_{i+1,j}^{n}-T_{i-1,j}^{n}}{2h_{1}} \end{array}$$

**Figure 3:**
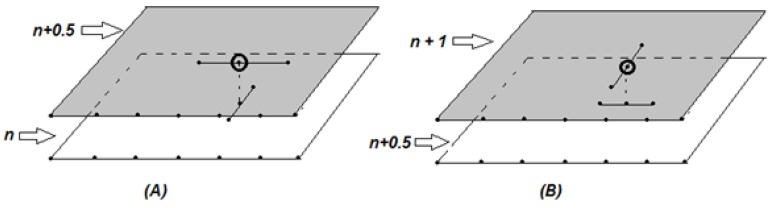
Approximation patterns in the inner points for problems *(**A**)* and *(**B**)*. In the left figure for *(**A**)*—the planes are $\Omega^{n},\Omega^{n+1/2}$ ; for *(**B**)*—the planes are $\Omega^{n+1/2}$, $\Omega^{n+1}.$

Several linear systems are solved in order to handle (A) and (B). For the problem (A) each system consists of equations for the points ${\left\{T_{i,j}^{n+\frac{1}{2}}\right\}}_{i=\bar{0,N}}$ along $\Omega^{n}$ (*j* and *n* are fixed, Figure 3). For the problem (B) such a system consists of equations concerning the points ${\left\{T_{i,j}^{n+1}\right\}}_{i=\bar{0,M}}$ (*i* and *n* are fixed, Figure 3). The obtained linear systems are three-diagonal and are solved via the Thomas method. The numerical scheme developed is absolutely stable and implicit in terms of *τ* due to the definition of problems (A) and (B) proposed in the non-stationary mathematical model.

The thermal conductivity *λ* and the specific heat capacity $C_{p}$ for each investigated metal are implemented in the presented model as functions of the temperature (see 2.1, Table 1). When solving *(A)* and *(B)*, temperature distribution from the previous iteration is used for evaluation of *λ* and $C_{p}$.

## 4. Optimization Model and Different Criteria

**Figure 4:**
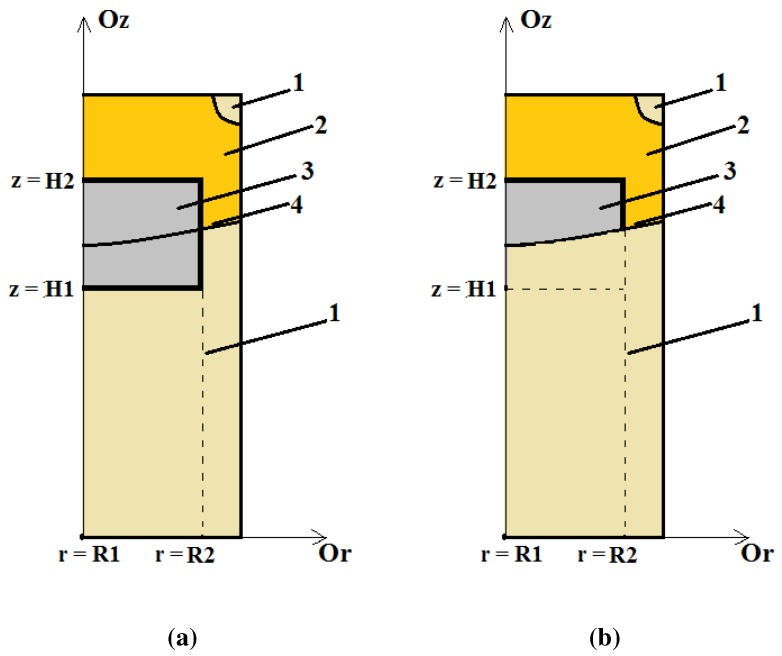
Geometrical characteristics on a half of the vertical ingot cross-section, $R_{1}=0;$ 1—solid metal; 2—liquid metal; 3—projection of *D* onto (r,z) plane; 4—liquid/solid metal contour. (**a**) $D=\left[R_{1},R_{2}\right]\times \left[H_{1},H_{2}\right]\times \left[F_{1},F_{2}\right]\subseteq \Omega$; (**b**) $D=\left[R_{1},R_{2}\right]\times \left[H_{1},H_{2}\right]\times \left[F_{1},F_{2}\right]\cap \left\{\left(r,z,t\right)|T\left(r,z,t\right)\geq T_{melt}\right\}$.

**Figure 5:**
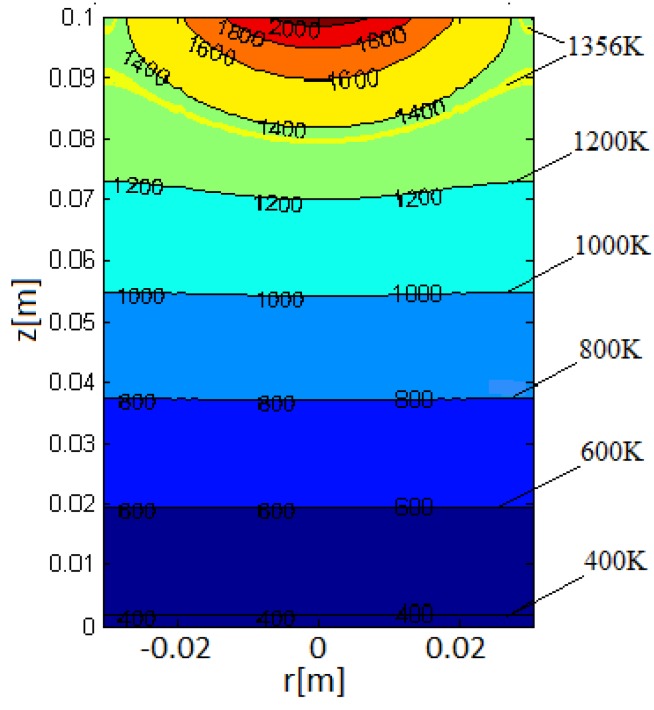
Temperature distribution in vertical cross-section of Cu cylindrical sample. The solid yellow curve ($T_{melt}=1356$ K) is the liquid/solid boundary in the metal.

**Figure 6:**
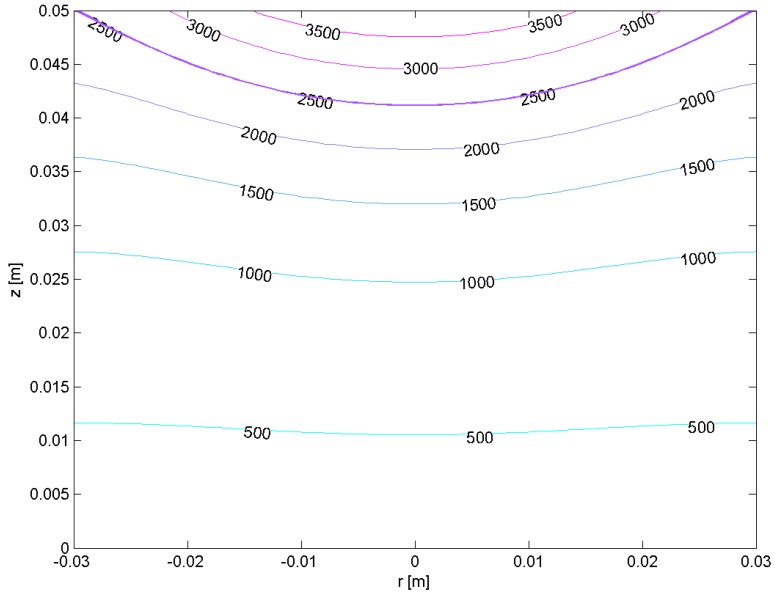
Temperature distribution in a vertical cross-section of Hf ingot (H= 50 mm, R= 30 mm) at the 295th second of EBM process for $P_{b}=$ 18 kW, $r_{b}=$ 18 mm, $T_{melt}=$ 2506 K.

The knowledge of the geometry of the crystallization front (liquid/solid boundary) is very important for studying and optimizing the quality of the obtained pure metal after the EBMR process. The flatness of the liquid/solid contour is directly connected to the quality of the structure of the obtained metal. The flat crystallization front permits the formation of vertical dendrite structure and uniform impurities’ displacement toward the ingot top surface (Figure 4, Figure 5 and Figure 6). When the liquid/solid boundary is deep in the center part of the metal block and shallow at the periphery of the cast ingot, a non-uniform structure along the ingot radius will be formed. The flatness of the temperature lines in a vertical cross-section of the cylindrical metal ingot for a fixed moment of time depends on the values of $\frac{\partial T}{\partial r}$. For fixed moment of the heating $t=t_{f}$ and height $z=z_{f},T\left(r;z_{f};t_{f}\right)$ is a decreasing function of the variable *r* and $\frac{\partial T}{\partial r}\leq 0$. Our aim is to maximize the flatness of the contour liquid metal/ solid metal.

### 4.1. Different Criteria

In this paragraph we discuss different criteria aiming to obtain maximum flatness of the crystallization front shape. One approach is to minimize the average of $\left(-\frac{\partial T}{\partial r}\right)$ over an area that includes the liquid/ solid boundary. Then, the criterion is:

(25) $$\begin{array}{c} A\left(P_{b},r_{b},V\right)=-\underset{D}{\;\int \int \int \;}\mu \left(r,z,t\right)\frac{\partial T}{\partial r}\left(r,z,t\right)drdzdt\to min \end{array}$$

where *T* is the temperature field determined by the solution of Equations (2) and (7–11) and μ(r,z,t) is a weight function. The control variables are the casting velocity *V*, the beam power $P_{b}$ and the beam radius $r_{b}$·$P_{b}$ is connected with the heating beam energy distribution $P_{surf}$. For a fixed heating time $t_{f}$, the beam energy distribution $P_{surf}\left(r,t_{f}\right)$ is a Gaussian-like function and:

(26) $$\begin{array}{c} \int_{0}^{R}P_{surf}\left(r,t_{f}\right)dr=P_{b} \end{array}$$

Minimization of Equation (25) can be realized over an area *D* that includes the crystallization front (the molten pool contour, Figure 4a):

(27) $$\begin{array}{c} D=\left[R_{1},R_{2}\right]\times \left[H_{1},H_{2}\right]\times \left[F_{1},F_{2}\right]\subseteq \Omega \end{array}$$

In a more complicated case *D* (Figure 4b) can be defined as:

(28) $$\begin{array}{c} D=\left[R_{1},R_{2}\right]\times \left[H_{1},H_{2}\right]\times \left[F_{1},F_{2}\right]\cap \left\{\left(r,z,t\right)|T\left(r,z,t\right)\geq T_{melt}\right\} \end{array}$$

Another approach is to minimize the mean curvature of the curve $\Gamma (t_{f})$, corresponding to the liquid/ solid boundary (Figure 4) at a fixed moment of heating $t_{f}$:

(29) $$\begin{array}{c} \int_{0}^{S}\mu_{2}\left(s\right)||\frac{d\Gamma (t_{f})}{ds}\left(r(s),z(s)\right)\left|\right|ds\to min \end{array}$$

where:

(30) $$\begin{array}{c} \Gamma \left(t_{f}\right)=\left\{\left(r,z\right)|T\left(r,z,t_{f}\right)=T_{melt}\right\} \end{array}$$

or to minimize the mean curvature over a heating time interval:

(31) $$\begin{array}{c} \int_{t_{1}}^{t_{2}}\mu_{1}\left(t\right)\int_{0}^{S}\mu_{2}\left(s\right)||\frac{d\Gamma (t)}{ds}\left(r(s),z(s)\right)\left|\right|dsdt\to min \end{array}$$

For Γ(t), *s* is a natural parameter for the curve and $\mu_{1},\mu_{2}$ are weight functions. The weight functions in Equations (25) and (31) can be defined in different ways taking into account the peculiarities of the electron beam melting process of the investigated metal. The used weight function in the paper is constant.

### 4.2. Discretization of the Criteria

The proposed criteria for achieving flatness of the crystallization front shape can be discretized synchronically to the proposed numerical scheme for the heat model. For the criterion (25) and *D*, described by Equation (27) or Equation (28), the multidimensional trapezoidal rule can be used. In the case of Equation (27) lets denote:

(32) $$\begin{array}{c} S_{i,j}^{n}=\mu \left(t_{n}\right)\frac{T_{i,j}^{n}-T_{i-1,j}^{n}}{h_{1}}+O\left(h_{1}\right) \end{array}$$

where ${\left\{T_{i,j}^{n}\right\}}_{i=\bar{1,N},j=\bar{1,M}}^{n=\bar{1,P}}$ is the discrete temperature field calculated by the modified Pismen-Rekfort scheme. The subnet of $W_{h_{1},h_{2},\tau }$ which is used to discretize the criterion is:

(33) $$\begin{array}{cc} & W_{h_{1},h_{2},\tau }^{1}=\left\{\left(r_{i},z_{j},t_{n}\right)|r_{i}=ih_{i},z_{j}=jh_{2},t_{n}=n\tau ;i=\bar{N_{1},N_{2}},j=\bar{M_{1},M_{2}},n=\bar{P_{1},P_{2}}\right\} \end{array}$$

In Equation (33), $N_{i}$ corresponds to $R_{i}$, $M_{i}$ corresponds to $H_{i}$, and $P_{i}$ corresponds to $F_{i}$, i=1,2. Formula (34) presents the approximation of Equation (25):

(34) $$\begin{array}{ccc} -\frac{8}{h_{1}h_{2}\tau }A & = & S_{N_{1},M_{1}}^{P_{1}}+S_{N_{1},M_{1}}^{P_{2}}+S_{N_{1},M_{2}}^{P_{1}}+S_{N_{1},M_{2}}^{P_{2}}+S_{N_{2},M_{1}}^{P_{1}}+S_{N_{2},M_{1}}^{P_{2}}+S_{N_{2},M_{2}}^{P_{1}}+S_{N_{2},M_{2}}^{P_{2}}+\\ & + & 2\left\{\begin{array}{c} \sum_{i=N_{1}+1}^{N_{2}-1}\left[S_{i,M_{1}}^{P_{1}}+S_{i,M_{2}}^{P_{1}}+S_{i,M_{1}}^{P_{2}}+S_{i,M_{2}}^{P_{2}}\right]+\\ \sum_{j=M_{1}+1}^{M_{2}-1}\left[S_{N_{1},j}^{P_{1}}+S_{N_{2},j}^{P_{1}}+S_{N_{1},j}^{P_{2}}+S_{N_{2},j}^{P_{2}}\right]+\\ \sum_{n=P_{1}+1}^{P_{2}-1}\left[S_{N_{1},M_{1}}^{n}+S_{N_{1},M_{2}}^{n}+S_{N_{2},M_{1}}^{n}+S_{N_{2},M_{2}}^{n}\right] \end{array}\right.\\ & + & 4\left\{\begin{array}{c} \sum_{i=N_{1}+1}^{N_{2}-1}\sum_{j=M_{1}+1}^{M_{2}-1}\left[S_{i,j}^{P_{1}}+S_{i,j}^{P_{2}}\right]+\\ \sum_{j=M_{1}+1}^{M_{2}-1}\sum_{n=P_{1}+1}^{P_{2}-1}\left[S_{N_{1},j}^{n}+S_{N_{2},j}^{n}\right]+\\ \sum_{n=P_{1}+1}^{P_{2}-1}\sum_{i=N_{1}+1}^{N_{2}-1}\left[S_{i,M_{1}}^{n}+S_{i,M_{2}}^{n}\right] \end{array}\right.\\ & + & 8\sum_{i=N_{1}+1}^{N_{2}-1}\sum_{j=M_{1}+1}^{M_{2}-1}\sum_{n=P_{1}+1}^{P_{2}-1}S_{i,j}^{n} \end{array}$$

For discrete calculation of the criteria (29) and (31), the curve Γ is approximated by a set of points. Then the curve can be interpolated by linear regression method or some interpolation method.

After the criterion discretizing and choosing the optimization variables (the input power $P_{b}$, the e-beam radius $r_{b}$, the casting velocity *V*, *etc.*) for solving the defined optimization problem, an algorithm is needed. Heuristic methods (clustering optimization technique, genetic algorithms, *etc.*) are appropriate because the dependence of the criteria on the control variables is implicit.

## 5. Results and Discussion

The whole developed process tool of modeling, simulation and optimization of the process of EBMR of metals can be summarized as follows:

1. Theoretical mathematical modeling of the heating processes during EBMR of metals and alloys, describing the equations of the model;
2. Construction of a modified Pismen-Rekford numerical scheme of the model;
3. Development of a software for model simulation via the numerical scheme;
4. Verification of the model;
5. Development of analytical criteria for optimization of the quality of the obtained new materials by achieving flatness of the crystallization front shape;
6. Discretization of the criteria synchronically to the numerical discretization of the model and computer implementation;
7. Choice of heuristic optimization techniques and synchronization of all developed computer programs;
8. Tests using different combinations of criteria, control variables and optimization techniques.

A corresponding computer program, based on the proposed time–dependent heat model, is developed for the study of the thermal processes at EBMR of metals. In this paper, results for EBMR of copper and hafnium are presented and discussed. The temperature variations of the thermal conductivity *λ* and the heat capacity $C_{p}$ for Cu and Hf are estimated using experimental data [19,21]. The obtained dependencies are presented in Table 1.

**Table 1:** Material characteristics used in calculations.

Parameter	Hf	Cu
$T_{melt}$, K	2506	1356
$\lambda ,[\frac{\text{W}}{\text{m}\cdot \;\text{K}}]$	$\left\{\begin{array}{cc} & \;25.17,\;T<293\;\text{K}\\ & \;26.05-\frac{1.54}{10^{2}}T+\frac{8.95}{10^{6}}T^{2}+\frac{8.46*10^{2}}{T},\;293\;\text{K}\leq T\leq 2000\;\text{K}\\ & \;31.3409,\;T>2000\;\text{K} \end{array}\right.$	$\left\{\begin{array}{cc} & \;439.1-0.0937T,\;T<1500\;\text{K}\\ & \;320,\;T>1500\;\text{K} \end{array}\right.$
$C_{p},\left[\frac{\text{W}\cdot \text{s}}{\text{g}\cdot \text{K}}\right]$	$\left\{\begin{array}{cc} & \;0.14225,\;T<293\;\text{K}\\ & \;\frac{23.347+8.004*10^{-3}*T-1.058*10^{4}*T^{-2}}{178.49},\;293\;\text{K}\leq T\leq 2000\;\text{K}\\ & \;0.220474,\;T>2000\;\text{K} \end{array}\right.$	$\left\{\begin{array}{cc} & \;0.388,\;T<293\;\text{K}\\ & \;0.36+\frac{9.96}{10^{4}}T,\;293\;\text{K}\leq T\leq 1356\;\text{K}\\ & \;0.494,\;T>1356\;\text{K} \end{array}\right.$

Simulation in copper (Cu) cylindrical ingots (2R=60 mm, H= 100 mm) for e-beam powers $P_{b}=$ 20 kW, beam radius $r_{b}=12$ mm and for 10 min heating time at V= 0 mm/min is made. The temperature fields are computed and the profiles of the molten metal pool are investigated. In Figure 5a calculated temperature field in a vertical cross-section of the ingot at the 10th minute of the heating is shown. The solid yellow curve represents the temperature level contour corresponding to the copper melting temperature $T_{melt}=$ 1356 K.

A series of experiments realizing different technological regimes are performed in our laboratory “Physical problems of electron beam technologies", Institute of electronics, Bulgarian Academy of Sciences, using ELIT 60 equipment for EBMR. Liquid pool shape variations (experimental and simulation data) in copper samples (2R= 60 mm, H= 50 mm) *vs.* the refining time are shown in Table 2 at heating with different e-beam powers of 10 kW and 15 kW, $r_{b}=$ 12 mm and V=0 mm/min. The obtained experimental results, investigated (after EMBR) by a metallographic etching method, are compared with simulation data, obtained by the presented heat model. The results about the geometry of the crystallization front shape—diameters $d_{m}$ and depths $h_{m}$ of the liquid metal pool are shown in Table 2. A good correspondence between calculated and experimentally obtained shapes of the crystallization front is observed.

**Table 2:** Experimental and simulation data about the geometry of the molten pool during EBMR of copper. E—experimental data; S—simulation data.

$P_{b},$ kW	Characteristics	at 10th min		at 15th min		at 20th min		at 30th min	
		E	S	E	S	E	S	E	S
10	$d_{m}$, mm	50	45	60	60	60	60	60	60
	$h_{m}$, mm	13	12	14	14	17	15	19	15
15	$d_{m}$, mm	60	60	60	60	60	60	60	60
	$h_{m}$, mm	20	20	22	21	19	20	20	20

Using the developed simulation tool important data about the geometry of the liquid metal pool, heat streams through the boundaries, temperature fields in vertical and/or horizontal cross-sections of the cylindrical ingots during the EBMR process are obtained. Calculated and experimentally obtained crystallization front shapes are compared and a good correspondence is observed. Conclusions concerning the influence of a variety of regime parameters (such as e-beam radius, beam power, casting velocity, *etc*.) are made based on the simulation results [13,15,24]. They can be used for optimization of the heat streams according to the process requirements and for choosing proper process conditions. They are used in the developed optimization approach concerning the flatness of the crystallization front shape.

Using the developed tools and corresponding computer programs numerical experiments for EBMR of hafnium are made. The ingot’s dimensions are H= 50 mm and R= 30 mm, the total heating time is 6 min, V= 0 mm/min. The radius of the e-beam is 18 mm and the beam power is 18, 20, 22, 24 kW. Simulation results for the heat transfer processes are investigated. It is observed that for each beam power the molten pool is mostly flat at the moment before the liquid pool reaches *G2* boundary for a first time (Table 3). For all the beam powers the molten pool periodically reaches and withdraws *G2*.

**Table 3:** First moment of contact between the molten pool and *G2* boundary.

No.	$P_{b}$, kW	Time of first contact, s
1	18	300
2	20	230
3	22	180
4	24	150

Using the obtained simulation results, one dimensional cluster optimization technique for EBMR of Hf is performed. The control variable is the beam power $P_{b}$. The chosen criterion is Equation (25) with a constant weight function. The investigated range for the beam power is [18 kW, 24 kW] and the cluster analysis is made for three different areas *D*: [0,R]×[0.7H,0.9H]×[0.2F,F]; [0,R]×[0.7H,0.9H]×[0.2F,0.6F] and [0.2R,0.5R]×[0.7H,0.9H]×[0.2F,0.6F]. The choice of the areas *D* is based on the results obtained by the heat model (Figure 4 and Figure 5). The results obtained by the one dimensional cluster optimization show that for all three different areas *D*, the flattest molten pool (according to the criterion (25)) is observed for $P_{b}=$ 18 kW (Figure 6). So a possible technological suggestion is that for the investigated technological regimes a lower e-beam power (18 kW) has to be kept.

The cluster optimization technique is applied to optimize the technological regime for electron beam melting of titanium. The criterion (25) is minimized for $P_{b}=$ 11 kW [25]. This result is compared and coincides to the value obtained by experimental data and statistical method [26]. An important advantage of the optimization scheme proposed is that experimental data for chemical analysis of the impurities’ concentrations for different technological regimes are not needed. The developed optimization approach can be applied to control and suggest proper technological parameters for electron beam melting of different metals for improving the quality of the obtained new materials.

## 6. Conclusions

A time-dependent thermal mathematical model, corresponding numerical method and computer program for simulation of heat transfer in metal ingots during EBMR are developed and presented. The heat model is 3D axis-symmetric and different heat transfer mechanisms through the boundaries are assumed. The developed numerical scheme is absolutely stable and implicit in terms of the time. The non-stationary heat model is programmed and corresponding computer software based on the model is also developed. The mathematical model allows acquiring important for the practice data and dependencies, which are otherwise difficult to obtain through EBMR experimental study (such as liquid pool geometry, energy losses, temperature distributions in metal ingots, *etc.*). The developed tool is employed to predict the evolution of the temperature, streams and crystallization front shape during electron beam melting and refining of different metals (hafnium, copper). Calculated results are compared to experimental data and good correspondence is observed. In order to optimize the EBMR process, different criteria, concerning the flatness of the crystallization front shape, are proposed and discussed. Analytical optimization problems are formulated, discretized using the heat model and solved by a cluster optimization method. The developed computational modeling and optimization tools and results obtained are important and give opportunity for better understanding, studying and optimizing the EBMR process and quality improvement of the purified materials produced by this expensive modern technology.
